# Broccoli-derived nanovesicles protect against UVB-induced skin photoaging via integrated transcriptomic and proteomic reprogramming of redox and extracellular matrix homeostasis

**DOI:** 10.3389/fcell.2026.1806671

**Published:** 2026-06-05

**Authors:** Kunjie Li, Songfa Lin, Yanwei Xiao, Pengjun Zhou, Niu Xiang, Bo Cheng

**Affiliations:** 1 Department of Dermatology, The First Affiliated Hospital of Fujian Medical University, Fuzhou, Fujian, China; 2 Department of Dermatology, The Second Affiliated Hospital of Fujian Medical University, Quanzhou, Fujian, China; 3 Department of Dermatology, Aoti Campus of the First Affiliated Hospital, Fujian Medical University, Fuzhou, China; 4 Institute of Dermatology and Venereology, Fujian Medical University, Fuzhou, Fujian, China; 5 Key Laboratory of Skin Cancer of Fujian Higher Education Institutions, The Fujian Medical University, Fuzhou, Fujian, China

**Keywords:** broccoli-derived nanovesicles, extracellular matrix remodeling, multi-omics, oxidative stress, skin photoaging

## Abstract

Ultraviolet B (UVB) radiation is a major environmental factor driving skin photoaging by disrupting redox balance, activating inflammatory cascades, and accelerating extracellular matrix (ECM) degradation. Although plant-derived nanovesicles have emerged as promising bioactive delivery systems, their regulatory mechanisms in skin homeostasis remain incompletely understood. Here, we investigated the protective effects and molecular mechanisms of broccoli-derived nanovesicles (BDNVs) against UVB-induced skin photoaging *in vitro* and *in vivo*. BDNVs effectively reduced intracellular reactive oxygen species (ROS) accumulation, restored proliferation and migration capacity of human dermal fibroblasts (HDFs), and attenuated inflammatory cytokine expression and matrix metalloproteinase activation. In a UVB-induced mouse model, topical administration of BDNVs alleviated epidermal hyperplasia and preserved dermal collagen integrity. To elucidate the underlying mechanisms, integrated transcriptomic and proteomic analyses were performed on dorsal skin tissues from PBS- and BDNVs-treated mice. Multi-omics profiling revealed coordinated reprogramming of gene and protein expression associated with oxidative stress response, inflammatory signaling, and extracellular matrix organization. Enrichment analyses identified significant modulation of redox regulatory pathways, cytokine-mediated signaling, and ECM remodeling networks, suggesting that BDNVs act through multi-layered regulatory mechanisms rather than a single molecular target. Collectively, our findings demonstrate that BDNVs mitigate UVB-induced skin photoaging by restoring redox–inflammatory–ECM homeostasis through coordinated transcriptomic and proteomic regulation. This study provides mechanistic insight into plant-derived nanovesicles as natural nano-biomaterials for skin regenerative and anti-photoaging applications.

## Introduction

Photoaging, a complex biological process induced by chronic exposure to ultraviolet (UV) radiation, is one of the major causes of structural and functional deterioration of skin (Bang et al., 2021). Unlike intrinsic aging, photoaging has a distinct environmental etiology, marked by excessive accumulation of reactive oxygen species (ROS), dysfunctional dermal fibroblasts, increased collagen degradation, and disruption of extracellular matrix architecture (Wang et al., 2021; Aleksandrova et al., 2021). These pathological changes not only weaken the mechanical strength and barrier function of the skin but also significantly impair its ability to repair and renew itself, thereby accelerating the development of senescence-associated cutaneous phenotypes (Cao et al., 2022).

Human dermal fibroblasts, as key effector cells, are crucial in maintaining skin homeostasis and structural integrity (Quan et al., 2015). Exposure to UV radiation can trigger oxidative stress responses, disrupt the intracellular redox balance, inhibit fibroblast proliferation and migration, promote the development of cellular senescence phenotypes, and accelerate the degradation of collagen and extracellular matrix proteins through the activation of multiple proteolytic pathways (Zhao et al., 2022; Kim et al., 2020). Despite the development of various antioxidants, functional small molecules, and bioactive materials aimed at mitigating photoaging processes, these strategies still present significant limitations. These include issues with biostability, tissue penetration capacity, cell-targeting specificity, and long-term safety profiles, which prevent systematic regulation of the cutaneous microenvironment (Sauler et al., 2022; Anderson et al., 2019).

Although various anti-photoaging approaches have been proposed, their potential side effects and sustainability concerns have driven continuous exploration for novel intervention strategies that are environmentally friendly and safe (Wan et al., 2020). In recent years, extracellular nanovesicles of natural origin have garnered significant interest due to their distinct structural attributes and biological functions (Zeng et al., 2021). Nanovesicles derived from plants are a subset of these naturally secreted nanoscale membranous structures produced by plant cells. They possess the ability to stably transport a variety of bioactive components and play pivotal roles in cross-species biological regulation (Qin et al., 2017). When contrasted with synthetically produced nanomaterials, plant-derived nanovesicles demonstrate superior biocompatibility, reduced immunogenicity, and potential for scalable production. This positions them as promising candidates for applications in skin aging intervention (Chen et al., 2022; Yang et al., 2020).

However, although existing studies have suggested that plant-derived nanovesicles have potential biological activities in anti-inflammatory, antioxidant and tissue protection effects (Kim et al., 2022; Yang et al., 2023; Kim et al., 2022; Marinaro et al., 2019), their systematic mechanisms of action on skin photoaging are still not well understood. In particular, whether these nanovesicles can regulate oxidative stress, fibroblast function and dermal structure remodeling at both the cellular and tissue levels simultaneously is still lack of direct and systematic experimental evidence. Moreover, for this highly complex pathological process of photoaging, most related researches are still limited to the observation of a single indicator or local effect, and there is no comprehensive analysis from phenotypic changes to molecular network remodeling (Wu et al., 2015; Wang et al., 2022; Liu et al., 2023).

In this study, we focused on broccoli-derived nanovesicles (BDNVs) and systematically evaluated their biological effects in UVB-induced skin photoaging. Through comprehensive physicochemical characterization of BDNVs combined with human dermal fibroblast models and a hairless mouse photoaging model, we investigated their regulatory effects on photoaging-associated pathological alterations at the levels of cellular function, tissue architecture, and molecular signaling. Importantly, to elucidate the underlying regulatory mechanisms in a systems-level manner, we further performed integrated transcriptomic and proteomic analyses of skin tissues following BDNVs treatment. Multi-omics profiling enabled the identification of coordinated gene–protein expression changes and regulatory networks associated with redox homeostasis, inflammatory signaling, and extracellular matrix remodeling. By linking phenotypic improvement with molecular reprogramming, this study provides mechanistic insight into how plant-derived nanovesicles modulate skin homeostasis and establishes a multi-layered research framework for the development of natural nanovesicle-based anti-photoaging strategies.

## Materials and methods

### Extraction and characterization of BDNVs

BDNVs were isolated by ultracentrifugation. Fresh broccoli was first homogenized in pre-chilled PBS, and then centrifuged at 1,000 *g* for 10 min, 5,000 g for 30 min, and 10,000 g for 1 h to remove cell debris and large organelles stepwise. The supernatant was filtered through a 0.22 μm membrane filter, followed by ultracentrifugation at 100,000 g for 2 h. The resulting pellet was collected and resuspended to obtain purified nanovesicles. Then the morphology, particle size distribution and surface potential of obtained products were characterized by transmission electron microscopy (TEM), nanoparticle tracking analysis (NTA) and Zeta potential assay, respectively. Lipidomic analysis was further performed on the purified nanovesicles.

### Cell culture

Human dermal fibroblasts (HDFs) were purchased from Wuhan Pnine Life Science and Technology Co., Ltd. (Wuhan, China). The cells were cultured in DMEM medium supplemented with 10% fetal bovine serum (FBS) and 1% penicillin–streptomycin solution at 37 °C in a humidified atmosphere of 5% CO_2_. When the cells reached approximately 80% confluence, they were dissociated by 0.25% trypsin for subculturing. All experiments used cells in the logarithmic growth phase.

### Proliferation assay

Cell proliferation ability was evaluated using BeyoClick™ EdU-488 Cell Proliferation Assay Kit (Beyotime, China). After treatment, cells in each experimental group were incubated with 10 μM EdU for 2 h. Then the cells were harvested by trypsin digestion and fixed with 4% paraformaldehyde. According to the manufacturer’s instructions, cell permeabilization and Click reaction staining were performed successively. The cell proliferation was measured by flow cytometry. FSC and SSC parameters were used to gate out cell debris, and singlet gating was applied to exclude cell aggregates. Finally, EdU signals were collected and analyzed in Alexa Fluor 488 fluorescence channel, and the percentage of EdU positive cells represented the level of cell proliferation.

### Determination of ROS content

Intracellular reactive oxygen species (ROS) levels were measured using a ROS assay kit (Beyotime, China). HDFs subjected to different treatments were seeded at prede-termined densities and cultured until adherence. The medium was then replaced with serum-free culture medium containing 10 μM DCFH-DA probe, followed by incubation in the dark at 37 °C for 30 min. After incubation, cells were gently washed three times with ice-cold PBS to completely remove non-incorporated probes, then harvested via digestion for flow cytometry analysis. Flow cytometric detection was performed on a BD flow cytometer. Cellular debris was excluded through gating based on forward scatter (FSC) and side scatter (SSC), while single-cell gates were applied to eliminate cell aggregates. ROS levels were determined by detecting DCF fluorescence signals collected in the FITC channel, with results expressed as mean fluorescence intensity (MFI).

### Migration determination

Cell migration was assessed using a transwell chamber assay. A 24-well transwell plate (pore size: 8 μm) was used. After UVB exposure and corresponding treatments for 24 h, HDFs were harvested, resuspended in serum-free DMEM at a density of 3 × 105 cells/mL. Complete medium containing 10% FBS (800 μL) was added to the lower compartment as a chemoattractant, while 200 μL of cell suspension was seeded into the upper compartment. Following incubation at 37 °C with 5% CO2 for 24 h, cells were fixed with pre-chilled 70% ethanol for 1 h and stained with 0.5% crystal violet for 20 min. Non-migrating cells on the top side of the membrane were carefully removed. Migrating cells on the bottom surface of the membrane were photographed under an inverted microscope (magnification ×100) by randomly selecting at least three fields per insert, and the number of migrating cells was counted using ImageJ software. The migratory ability was expressed as mean number of migratory cells per group.

### Scratch determination

HDFs were seeded in 6-well plates at a density of 2.5 × 105 cells/mL and scratch assay was performed when the cell confluence reached more than 90%. A straight line wound was created on the monolayer using a 200 μL sterile pipette tip perpendicular to the marking lines on the bottom of the plate. The detached cells were removed by washing three times with PBS gently, then the medium was replaced with serum-free culture medium. Images were captured under an inverted microscope (100 magnification) immediately (0 h) and 24 h after scratching, and at least three parallel wells were set for each experimental group. Scratch widths were measured using ImageJ software, and the migration rate of cells was calculated as follows: Migration rate (%) = [(Scratch width at 0 h - Scratch width at 24 h)/Scratch width at 0 h] × 100%.

### Western blot analysis

After treatment with BDNVs, human dermal fibroblasts were collected and lysed in a pre-cold lysis buffer to extract total protein. The concentration of all samples was determined by BCA Protein Assay Kit. Equal amounts of proteins were separated by SDS-PAGE electrophoresis and transferred onto polyvinylidene fluoride (PVDF) membranes. Membranes were blocked for 1 h at room temperature with 5% nonfat dry milk to prevent nonspecific binding. Subsequently, the membranes were incubated with specific primary antibodies against IL-1β, IL-6, MMP1, MMP9 and TNF-α according to the dilution ratio recommended by the manufacturer, washed and then incubated with corresponding secondary antibody. Protein bands were visualized using enhanced chemiluminescence (ECL) reagents, and band intensities were quantitatively analyzed using ImageJ software. Cells treated with PBS served as negative controls in the experiments.

### Establishment of photoaging animal model

The animal experimental protocol of this study was approved by the Animal Ethics Committee of Fujian Medical University (Approval No. [2023] FYFYEC-146). All experimental animals were acclimatized under standard conditions for 1 week before being used. Male nude mice aged 6–8 weeks were randomly divided into four groups, with seven mice in each group: blank group, model group, negative control group and BDNVs treatment group. The cumulative UVB dose over 15 exposures was 3 J/cm^2^. The minimal erythema dose was determined in a preliminary study by exposing mice to incremental UVB doses and evaluating erythema 24 h post-irradiation. With the exception of the blank group, all other groups received a fixed dose of UVB irradiation (200 mJ/cm^2^) on their depilated back skin every other day for a total of 15 times to establish a skin photoaging model. The concentration of BDNVs used for topical application (100 μg/mL) was selected based on a preliminary dose-response study in human dermal fibroblasts (HDFs), in which cells were treated with increasing concentrations of BDNVs (25, 50, 100, 200 μg/mL) following UVB exposure. The 100 μg/mL concentration exhibited the optimal protective effect in terms of cell viability, with no observable cytotoxicity. Therefore, this concentration was used for subsequent *in vivo* experiments to ensure consistency and comparability between *in vitro* and *in vivo* findings (Supplementary Figure S2). After the establishment of the model and continuous maintenance until the 29th day after modeling, topical administration was performed according to grouping treatment: BDNVs treatment group topically applied BDNVs solution (100 μg/mL) daily to the UVB-irradiated area for 14 consecutive days; the negative control group was treated with an equal volume of PBS as the vehicle control. Mice in the blank group did not receive UVB irradiation or any subsequent treatments.

### Histological analysis

After drug treatment, the dorsal skin tissues of nude mice were harvested for sub-sequent analysis. Tissue samples were fixed in 4% paraformaldehyde for 24 h, followed by gradient dehydration, paraffin-embedded and sectioned into consecutive slices (thickness: 4 μm). The sections were stained with hematoxylin-eosin (HE) and Masson’s trichrome for histological evaluation. In addition, the expression levels of IL-1β, IL-6, MMP1, MMP9 and TNF-α in skin tissues were measured using enzyme-linked immunosorbent assay (ELISA) kits according to the manufacturer’s instructions. All tissue sections were observed under a light microscope, and quantitative analyses of relevant indicators were performed using ImageJ software.

### Transcriptomics

Transcriptome sequencing was conducted on dorsal skin tissues from mice in both the PBS-treated and BDNVs-treated groups. Following the extraction of total RNA and successful passage of quality control assessments, cDNA libraries were generated and sequenced utilizing the Illumina high-throughput sequencing platform. The raw reads were subjected to quality filtering and subsequently aligned to the mouse reference genome. Uniform methodologies were employed for quantitative gene expression analysis. Differentially expressed genes (DEGs) were pinpointed based on established statistical criteria. Subsequently, GO functional annotation and KEGG pathway enrichment analyses were performed to shed light on the transcriptional regulatory mechanisms of BDNVs in counteracting cutaneous photoaging.

### Proteomics

Dorsal skin samples from the PBS treatment group and BDNVs treatment group were subjected to proteomic analysis. The liquid chromatography-tandem mass spectrometry (LC-MS/MS) technology was used for protein digestion, peptide purification and detection. Database search software such as MaxQuant was applied for protein identification and quantitative analysis. Then GO functional annotation and KEGG pathway enrichment analysis of differentially expressed proteins were performed to explore the potential regulatory mechanism of BDNVs in alleviating photoaging in mice skin.

### Data analysis

All data are expressed as the mean ± standard deviation (mean ± SD). Statistical analysis was performed using GraphPad Prism 10 software. Statistical significance be-tween groups is indicated by asterisks, where *p < 0.05, **p < 0.01, ***p < 0.001 and ****p < 0.0001. Each experiment was independently repeated at least three times.

## Results

### Characterization of BDNVs

**FIGURE 1:**
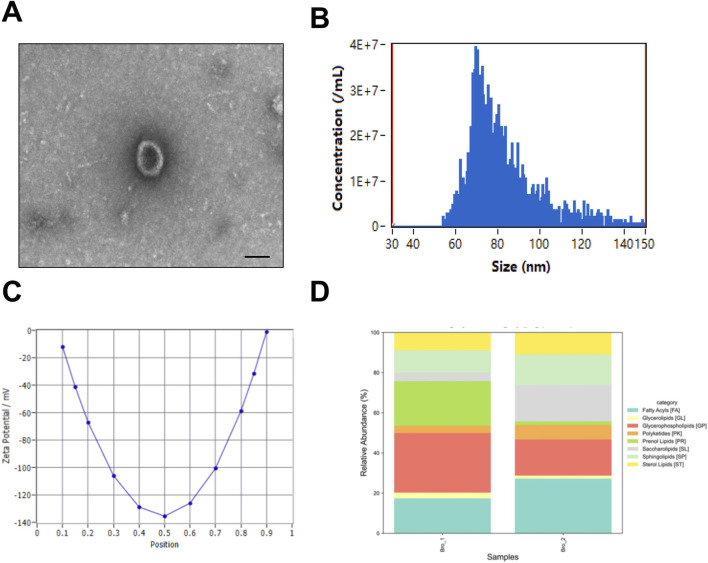
Characterization of BDNVs. **(A)** Transmission electron microscopy reveals a teacup-like morphology of BDNVs (scale = 100 nm). **(B)** Nanoparticle tracking analysis demonstrates a unimodal size distribution pattern of BDNVs. **(C)** Zeta potential analysis of BDNVs. **(D)** Lipidomic profiling of BDNVs.

BDNVs were isolated from freshly harvested broccoli using juice extraction and enzymatic hydrolysis, followed by differential ultracentrifugation. Transmission electron microscopy (TEM) revealed that the isolated BDNVs possess typical vesicular structures with a relatively uniform morphology and distinct bilayer membrane characteristics. Nanoparticle tracking analysis (NTA) showed a concentrated particle size distribution for BDNVs, with an average diameter of 77.8 nm (Figures 1A,B). The isolation yield was approximately 1.52 ± 0.18 mg protein per 100 g of fresh broccoli, as determined by BCA assay. Purity was further assessed by calculating the particle-to-protein ratio, which was 3.2 × 10^10^ particles/μg protein, indicating effective removal of non-vesicular contaminants. To evaluate batch-to-batch consistency, three independent isolations were performed. The resulting BDNVs exhibited similar characteristics in terms of particle size (coefficient of variation <10%), zeta potential (coefficient of variation <8%), and protein yield (coefficient of variation <12%), confirming the reproducibility of the isolation protocol.

Zeta potential analysis showed that BDNVs were negatively charged, with a mean value of −36.48 ± 0.93 mV (Figure 1C), indicating good colloidal stability.

Lipidomic analysis further revealed a diverse composition of lipid molecular components within BDNVs, reflecting their compositional complexity. The major identified lipid classes included fatty acyls, glycerolipids, glycerophospholipids, polyketides, and prenol lipids, indicating potential roles for BDEVsBDNVs in bioactive molecule delivery and the regulation of related biological processes (Figure 1D).

### BDNVs attenuate UVB-induced inhibition of proliferation in human dermal fibroblasts

To evaluate the protective effects of BDNVs against UVB-induced inhibition of fibroblast proliferation, flow cytometry was used to detect the proliferative status of HDFs in different treatment groups. Compared with Control group, the proportion of highly fluorescent cell population in FITC-H channel was significantly decreased after UVB irradiation, indicating that cell proliferation capacity was significantly inhibited. The distribution trend of fluorescence in UVB + PBS group was basically consistent with that in UVB group, suggesting that vehicle treatment alone could not improve the inhibitory effect of UVB on cell proliferation. However, the peak of high fluorescence type in UVB + BDNVs co-treatment group showed a significant right shift, and its distribution trend tended to be close to that of Control group, suggesting that BDNVs administration effectively alleviated the inhibitory effect of UVB on cell proliferation.

**FIGURE 2:**
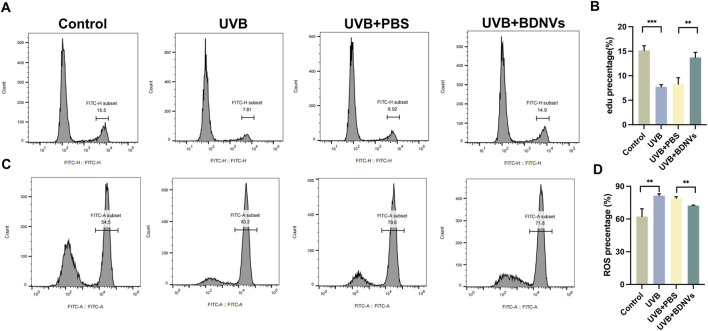
Effects of BDNVs on HDF proliferation and ROS generation following UVB irradiation. **(A)** Representative flow cytometry histograms showing cell proliferation of HDF cells after different treatments (FITC-H channel). **(B)** Quantitative analysis of cell proliferation based on flow cytometry data (**p < 0.01, ***p < 0.001). **(C)** Representative flow cytometry histograms illustrating intracellular ROS levels in HDF cells after different treatments (FITC-A channel). **(D)** Quantitative analysis of intracellular ROS levels based on flow cytometry data (**p < 0.01, ***p < 0.001).

Quantitative analysis showed that the percentage of positive cells in the UVB group was significantly lower than that in the control group (p < 0.001); compared with the UVB group, the proportion of positive cells in the UVB + BDNVs treatment group increased significantly (p < 0.001), and the recovery level was close to that in the control group. The above results indicated that BDNVs had a significant protective effect on UVB-induced inhibition of fibroblast proliferation (Figures 2A,B).

### BDNVs inhibit UVB-induced excessive accumulation of intracellular ROS

To further explore the antioxidant effects of BDNVs, flow cytometry was used to measure intracellular ROS levels in HDFs among different treatment groups. Compared with the Control group, UVB exposure significantly increased the fluorescence intensity in the FITC-A channel, and the histogram curve as a whole showed a rightward trend, indicating that there was a large amount of ROS accumulation in the cells. The fluorescence distribution pattern of the UVB + PBS group was similar to that of the UVB group, indicating that PBS treatment did not alleviate the oxidative stress response induced by UVB. In contrast, the fluorescence intensity of the UVB + BDNVs treatment group de-creased significantly, as indicated by the leftward regression of the histogram distribution and high overlap with the Control group, suggesting that BDNVs effectively inhibited UVB-induced ROS production. Statistical analysis revealed that the proportion of ROS-positive cells in the UVB group increased significantly compared with the Control group (p < 0.001). Of note, this proportion decreased significantly following UVB + BDNVs intervention compared with the UVB group (p < 0.001), thus confirming the ability of BDNVs to effectively counteract UVB-induced oxidative stress (Figures 2C,D).

### BDNVs enhance the migration and scratch wound healing capacity of HDFs following UVB-induced damage

**FIGURE 3:**
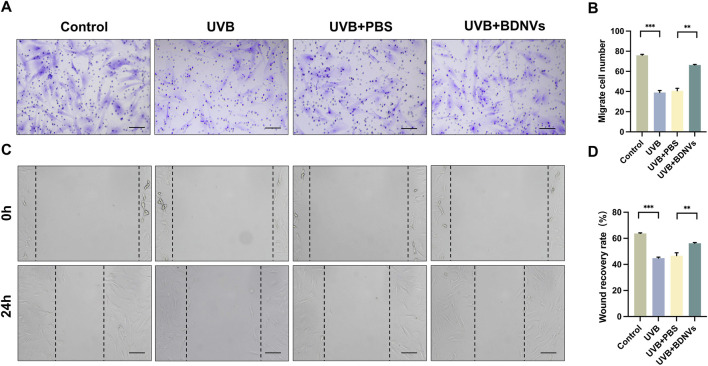
BDNVs enhance the migration and wound healing capacity of HDFs following UVB-induced damage. **(A)** Migration ability of HDFs after different treatments (scale = 100 μm). **(B)** Quantitative analysis of cell migration ability (**p < 0.01, ***p < 0.001). **(C)** Wound healing capacity of HDFs after different treatments (scale = 100 μm). **(D)** Quantitative analysis of cell wound healing capacity (**p < 0.01, ***p < 0.001).

To evaluate the effect of BDNVs on cell repair behavior after UVB injury, we further detected the migration ability and scratch wound healing of HDFs. The results of transwell chamber assay showed that compared with Control group, the number of cells migrating through matrigel membrane was significantly reduced under UVB irradiation, indicating that cell migration ability was significantly inhibited. However, in the co-treatment group (UVB + BDNVs), the number of migrated cells increased significantly, which was significantly higher than that in the UVB group, suggesting that BDNVs treatment effectively alleviated the inhibitory effect of UVB on cell migration (Figures 3A,B).

In order to further verify the effect of BDNVs on cell repair ability, scratch test was performed. All treatment groups had consistent initial scratch width at 0 h, thus eliminating experimental bias caused by initial differences. After 24 h of culture, quantitative analysis showed that the relative scratch closure rate of the UVB group decreased significantly compared with the control group (p < 0.001); while the scratch closure rate of the co-treatment group (UVB + BDNVs) increased significantly compared with the UVB group (p < 0.001), recovering to near control levels (Figures 3C,D).

These results indicate that BDNVs can not only reduce the inhibition of cell proliferation and oxidative stress induced by UVB, but also functionally enhance the migration and repair ability of HDFs, so as to promote the cellular repair process after UVB injury.

### BDNVs downregulate the expression of UVB-induced inflammatory response and extracellular matrix degradation-related proteins

**FIGURE 4:**
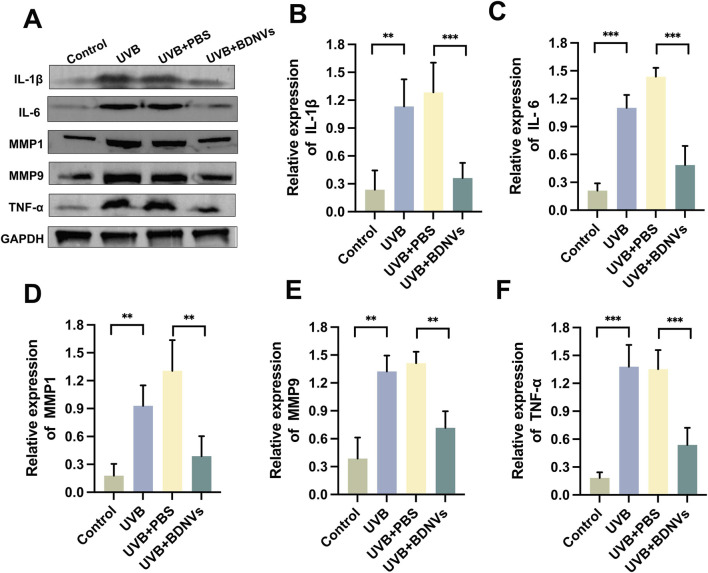
BDNVs inhibit UVB-induced expression of inflammatory factors and matrix metalloproteinases. **(A)** Protein expression levels of IL-1β, IL-6, TNF-α, MMP1, and MMP9 under different treatments. **(B)** Relative expression level of IL-1β. **(C)** Relative expression level of IL-6. **(D)** Relative expression level of MMP1. **(E)** Relative expression level of MMP9. **(F)** Relative expression level of TNF-α (**p < 0.01, ***p < 0.001).

UVB-induced skin injury is closely related to chronic inflammation and extracellular matrix degradation. To further explore the molecular protection mechanisms of BDNVs, we examined the expression levels of key inflammatory factors and MMPs by Western blot analysis. UVB irradiation significantly increased the protein levels of pro-inflammatory cytokines IL-1β, IL-6, TNF-α and matrix-degrading enzymes MMP1 and MMP9. After intervention with BDNVs, the expression levels of these proteins were reduced (Figure 4A).

Further quantitative analysis showed that compared with Control group, UVB irradiation significantly upregulated the expression of IL-1β, IL-6, TNF-α, MMP1 and MMP9 (all p < 0.001). As a control, UVB + PBS treatment failed to change this upregulation trend. Furthermore, co-treatment of UVB + BDNVs effectively reversed the abnormally high expression of all these proteins induced by UVB, which were significantly lower than those in UVB group (all p < 0.001). These results indicate that BDNVs not only functionally promote cell repair, but also exert anti-inflammatory effects and maintain extracellular matrix homeostasis at the molecular level by inhibiting UVB-triggered inflammatory signaling pathways and excessive expression of MMPs (Figures 4B–F).

### BDNVs demonstrated their efficacy in ameliorating photodamage in a UVB-induced aging mouse model

**FIGURE 5:**
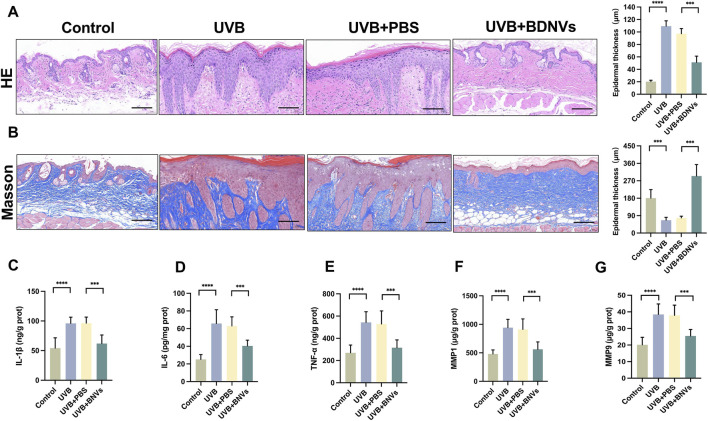
BDNVs attenuate UVB-induced photoaging damage in mouse skin *in vivo*. **(A)** Hematoxylin and eosin (HE) staining results of mouse skin sections from different treatment groups and quantitative analysis of epidermal thickness (scale = 100 μm). **(B)** Masson’s trichrome staining results of mouse skin sections from different treatment groups and quantitative analysis of dermal thickness. **(C)** Relative expression level of IL-1β. **(D)** Relative expression level of IL-6. **(E)** Relative expression level of MMP1. **(F)** Relative expression level of MMP9. **(G)** Relative expression level of TNF-α (***p < 0.001, ****p < 0.0001).

To verify the anti-photoaging effect of BDNVs *in vivo*, a UVB-induced skin photoaging model was established on hairless mice. The dorsal skin of each group showed different degrees of changes (Supplementary Figure S1). Histological analysis showed that UVB irradiation induced typical pathological changes related to skin photoaging: H&E staining showed significant thickening of the epidermis Masson’s trichrome staining showed reduced collagen fibers with disordered arrangement and significantly thinned dermal thickness. Consistent with the results of *in vitro* experiments, UVB + BDNVs treatment effectively improved these structural abnormalities, significantly inhibiting abnormal hyperplasia of the epidermis (Figure 5A, p < 0.001) and maintaining the thickness of the dermis and the structure of collagen (Figure 5B, p < 0.001).

*In vivo* molecular-level verification further confirmed the mechanism of action of BDNVs. ELISA results (Figures 5C–G) indicated that pro-inflammatory factors (IL-1β, IL-6, TNF-α) and extracellular matrix-degrading enzymes (MMP1, MMP9) were significantly increased in the skin tissues of the UVB group compared to those of the Control group. However, all these molecular markers were significantly inhibited by topical application of BDNVs in the UVB + BDNVs group, returning to near normal levels. These *in vivo* experimental data strongly proved that BDNVs could effectively resist UVB-induced skin photoaging at the whole animal level by inhibiting cutaneous inflammation response and collagen degradation, successfully realizing the transformation from cells to animals.

### Omics analysis

To explore the molecular regulatory mechanism of BDNVs in ameliorating UVB-induced skin photoaging, integrated transcriptome and proteomic analyses were performed on the dorsal skin tissues from mice in the PBS group and BDNVs treatment group.

**FIGURE 6:**
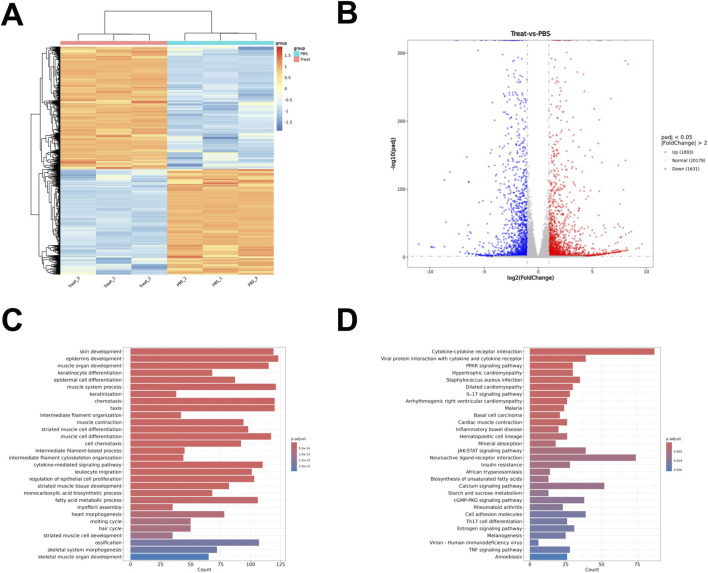
Transcriptomic analysis of mouse skin tissues before and after BDNVs administration. **(A)** Differential clustering heatmap after BDNVs treatment. **(B)** Volcano plot analysis before and after BDNVs application. **(C)** GO enrichment analysis of differentially expressed genes. **(D)** KEGG pathway enrichment analysis of differentially expressed genes.

Transcriptome analysis showed that BDNVs intervention significantly altered the overall transcriptional profile of skin tissues. A series of DEGs were screened according to predetermined statistical thresholds. Hierarchical clustering and principal component analysis revealed clear separation between the two samples, suggesting that BDNVs had a significant regulatory effect on the local microenvironment of the skin. GO functional annotation indicated that DEGs were mainly enriched in biological processes such as oxidative stress response, inflammatory regulation, extracellular matrix organization, cell proliferation and apoptosis regulation. KEGG pathway analysis further verified that related genes were significantly enriched in redox homeostasis regulatory pathways, inflammatory signaling cascades, and matrix remodeling-related signaling networks (Figure 6; Supplementary Table S1).

**FIGURE 7:**
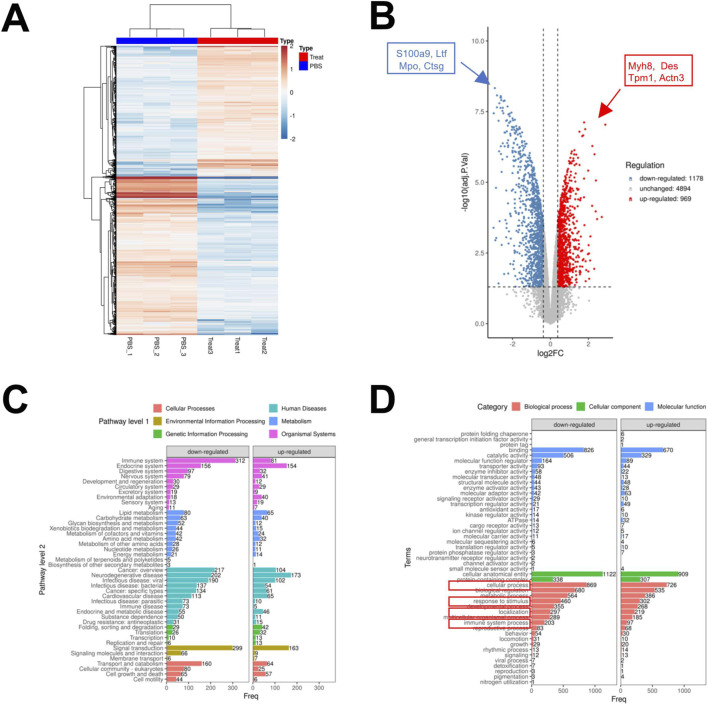
Proteomic analysis of mouse skin tissues before and after BDNVs administration. **(A)** Differential clustering heatmap after BDNVs treatment. **(B)** Volcano plot analysis before and after BDNVs application. **(C)** GO enrichment analysis of differentially expressed proteins. **(D)** KEGG pathway enrichment analysis of differentially expressed proteins.

Proteomic analysis also showed that BDNVs treatment significantly altered the protein expression profile of skin tissues. The screened DEPs (differentially expressed proteins) were mainly related to functional modules such as antioxidant defense systems, regulation of inflammatory factors, collagen metabolism and cytoskeletal remodeling. Among the significantly altered proteins, we observed that BDNVs treatment led to the upregulation of proteins involved in muscle contraction and cytoskeletal organization, including myosin-8 (Myh8), desmin (Des), tropomyosin (Tpm1), and α-actinin-3 (Actn3) Conversely, proteins associated with inflammatory responses and extracellular matrix remodeling were markedly downregulated, such as S100A9 (S100a9), lactotransferrin (Ltf), complement C3 (C3), myeloperoxidase (Mpo), and cathepsin G (Ctsg). These data suggest that BDNVs may exert protective effects by promoting cytoskeletal stability and suppressing inflammatory and tissue-degrading pathways. Pathway enrichment analysis indicated that these differentially expressed proteins were significantly involved in oxidative stress response, inflammatory signal transduction, extracellular matrix organization and degradation-related pathways (Figure 7; Supplementary Table S2). Further integrated transcriptome and proteomic analysis identified consistent variation trends across multiple core functional pathways at both molecular levels. Integrated analysis revealed consistent variation trends between transcriptomic and proteomic datasets. Specifically, inflammatory mediators such as S100a9, Ltf, C3, and Chil3 were consistently downregulated, while cytoskeletal and muscle contraction-related proteins including Des, Actn3, Tpm1, and Myh8 were consistently upregulated. Of note, many key molecules within the modules associated with oxidative stress regulation and inflammatory signaling modulation exhibited coordinated regulatory patterns at both transcriptional and protein levels, suggesting that BDNVs may exert therapeutic effects by synergistically acting on a multi-level molecular regulatory network to improve the microenvironment of UVB-induced skin injury. In addition, the expression changes of molecules associated with homeostasis of extracellular matrix suggested that BDNVs might play important roles in maintaining structural integrity of dermis and inhibiting stroma degradation.

## Discussion

This study systematically elucidates the multifaceted protective effects of BDNVs against UVB-induced skin photoaging and their underlying molecular regulatory mechanisms. Through a combination of functional experiments and multi-omics analysis at both transcriptomic and proteomic levels, we not only validated the biological effects of BDNVs in antioxidant defense, anti-inflammation, and matrix preservation but also dissected their potential regulatory networks from a systems biology perspective, thereby providing a more robust molecular foundation for the application of plant-derived nanovesicles in skin anti-aging research.

At the mechanistic level, the core function of BDNVs is primarily manifested by their potent antioxidant capacity. Excessive ROS generation induced by UVB irradiation represents the initial step of photoaging, which directly triggers lipid peroxidation, protein oxidative modifications, and DNA damage (Zhou et al., 2018). Our findings demonstrate that BDNVs significantly reduce intracellular ROS levels in HDFs. More importantly, integrated transcriptomic and proteomic analyses reveal that BDNV treatment markedly modulates multiple redox homeostasis-related signaling pathways, with key antioxidant defense molecules exhibiting coordinated changes at both transcriptional and translational levels. This suggests that BDNVs exert their effects through enhancing endogenous antioxidant defense systems rather than merely scavenging free radicals. Combined with lipidomic evidence showing enrichment of plant-derived antioxidant components (e.g., carotenoids, polyphenol derivatives), we propose that BDNVs may deliver bioactive lipids or signaling molecules to activate the Nrf2-mediated antioxidant regulatory network, thereby reprogramming cellular oxidative stress responses at the molecular level (Atamna et al., 2015; Zhang et al., 2016; Stanly et al., 2019).

In addition to the antioxidant effect, BDNVs modulate inflammatory signaling networks as another important mechanism for anti-photoaging. UVB-induced ROS can act as second messengers to activate inflammatory pathways such as NF-κB and MAPK, thereby inducing pro-inflammatory factors including IL-1β, IL-6, TNF-α, while promoting MMP expression, ultimately leading to collagen degradation and extracellular matrix structure damage (Shanmugam et al., 2017). Multi-omics analysis further revealed that genes and proteins related to inflammatory signal transduction and immune regulation were systematically regulated after BDNVs treatment. In particular, some core pathways showed coordinated changes at both transcriptional and protein levels, suggesting that BDNVs may interfere with key nodes where oxidative stress intersects with inflammatory signaling, thus achieving multi-target blockade of the pathological cascade reaction named “oxidative stress–inflammation amplification–matrix degradation” (Yepes-Molina et al., 2020; Qiao et al., 2018).

In terms of ECM homeostasis, the present study not only observed the restoration of collagen fiber structure at the histological level but also identified significant regulation of molecular networks related to ECM organization, collagen synthesis and matrix remodeling at multi-omics levels (Kim et al., 2022; Li et al., 2020). Transcriptomic and proteomic data demonstrated coordinated changes in the expression of multiple molecules involved in collagen metabolism, extracellular matrix organization and cytoskeletal remodeling, suggesting that BDNVs may maintain dermal structural stability by regulating the dynamic balance between matrix synthesis and degradation (Ly et al., 2023; Zhang et al., 2022; Yuan et al., 2020). This high consistency across molecular, cellular and tissue architecture levels enhanced the reliability of research conclusions.

At the functional level, BDNVs rescued the proliferation and migration abilities of HDFs, which constitute the cellular basis for tissue repair and regeneration (Zou et al., 2022; Shi et al., 2015; Liu et al., 2016; Monsuur et al., 2017). In animal models, BDNVs significantly ameliorated typical photoaging phenotypes including epidermal thickening and dermal collagen disruption. Multi-omics analysis provided molecular explanations for these phenotypic changes, allowing this study to establish an integrated evidence chain of “phenotypic validation-molecular pathway analysis-multi-omics integration.”

In contrast to conventional single antioxidants or synthetically engineered nanocarriers, BDNVs, being plant-derived natural nanovesicle systems, display multi-component synergistic regulation. Multi-omics analyses suggest that their photoprotective effects are not reliant on individual molecules or pathways but are instead accomplished through a comprehensive modulation by reshaping multi-level signaling networks. This “network-based regulatory paradigm” could signify a crucial advantage of plant-derived nanovesicles over traditional antioxidant approaches (Dong et al., 2019; Cui et al., 2022; Peng et al., 2022; Trac et al., 2019; Villasante et al., 2016).

The multi-layered regulatory mechanisms of BDNVs identified in this study align with and extend the current understanding of plant-derived nanovesicle biology. While previous studies have demonstrated that plant-derived nanovesicles possess antioxidant and anti-inflammatory properties, most have focused on individual pathways or isolated cellular responses. In contrast, our integrated multi-omics approach reveals that BDNVs orchestrate coordinated changes across multiple signaling modules simultaneously—including redox homeostasis, inflammatory cascades, and extracellular matrix remodeling—suggesting that their protective effects arise from network-level regulation rather than modulation of single molecular targets. This systems-level perspective distinguishes our work from prior reports and provides a more comprehensive mechanistic framework for understanding how plant-derived nanovesicles interact with skin tissues. Furthermore, the functional rescue of fibroblast proliferation, migration, and matrix integrity observed in our study bridges the gap between molecular pathway analysis and tissue-level outcomes, offering a more complete picture of the therapeutic potential of plant-derived nanovesicles in skin photoaging.

The mechanistic framework emerging from our study suggests that BDNVs exert their anti-photoaging effects through a coordinated tripartite mechanism involving redox homeostasis, inflammatory signaling, and ECM remodeling. At the molecular level, the transcriptomic and proteomic data reveal that BDNVs simultaneously upregulate antioxidant defense systems (e.g., Nrf2-mediated pathways) while downregulating pro-inflammatory mediators (e.g., NF-κB and MAPK signaling cascades) and matrix-degrading enzymes (e.g., MMP1, MMP9). This concurrent modulation of multiple signaling modules distinguishes BDNVs from conventional single-target antioxidants. Importantly, these molecular changes translate into functional recovery at the cellular level, as evidenced by restored fibroblast proliferation and migration, reduced ROS accumulation, and preserved dermal collagen architecture. Collectively, this integrated evidence chain—from molecular reprogramming to cellular function to tissue structure—provides a robust mechanistic basis for understanding how plant-derived nanovesicles protect against UVB-induced skin photoaging.

Nevertheless, this study has certain limitations. Firstly, although multi-omics analysis identified multiple potential key molecules and signaling pathways, the causal relationships have not been experimentally validated by specific inhibition or genetic intervention assays; Secondly, the specific bioactive components responsible for core regulatory functions in BDNVs and their relative contributions require further isolation and functional characterization; In addition, systematic investigations are needed regarding their *in vivo* distribution patterns, long-term safety profiles, and pharmacokinetic properties.

Future research should integrate deeper multi-omics data analysis with functional validation experiments to establish causal models of critical regulatory axes. Meanwhile, surface engineering or functional modifications could be employed to enhance targeted delivery efficiency while exploring their therapeutic potential in other oxidative stress-related skin diseases. We acknowledge that the current study did not include a PBS-only control group without UVB irradiation, which would theoretically provide additional baseline information regarding the effects of the solvent on normal skin. However, given that PBS is widely recognized as an inert buffer with no known biological activity, and that the UVB + PBS group already served as a vehicle control under pathological conditions, the absence of this group does not compromise the validity of our core conclusion that BDNVs exert therapeutic effects against UVB-induced photoaging. Future studies aiming to comprehensively evaluate the biosafety of plant-derived nanovesicles should include such a control. Besides, we acknowledge that the current *in vivo* study did not include a BDNVs-alone (no UVB) control group, which would have provided additional evidence for the safety of BDNVs on normal skin. However, our primary aim was to evaluate the therapeutic efficacy of BDNVs against UVB-induced photoaging. *In vitro* experiments demonstrated that BDNVs alone did not adversely affect the viability or proliferation of human dermal fibroblasts at concentrations up to 200 μg/mL, supporting their safety at the topical dose used *in vivo*. Future studies should incorporate a BDNVs-alone control to comprehensively evaluate the safety profile and potential biological effects of plant-derived nanovesicles in healthy skin.

## Conclusion

This study, which combines cell and animal model experiments with comprehensive transcriptomic and proteomic analyses, demonstrates that BDNVs effectively mitigate UVB-induced photoaging damage in the skin. This is achieved through the synergistic regulation of oxidative stress response, inflammatory signaling pathways, and extracellular matrix homeostasis networks. The multi-omics results further elucidate their molecular-level regulatory characteristics. As a naturally derived nanosystem that exhibits multi-component synergistic regulation, BDNVs offer a novel theoretical foundation and potential applications for the development of safe and efficient strategies for skin photoprotection and repair.

## Data Availability

The datasets presented in this study can be found in online repositories. The names of the repository/repositories and accession number(s) can be found in the article/Supplementary Material.
