# Supplementary material for: Broccoli-derived nanovesicles protect against UVB-induced skin photoaging via integrated transcriptomic and proteomic reprogramming of redox and extracellular matrix homeostasis
Source: Front Cell Dev Biol. 2026 Jun 5;14:1806671. doi: 10.3389/fcell.2026.1806671 (PMC13279611; doi:10.3389/fcell.2026.1806671)
Supplement: Supplementary file 2 [file DataSheet1.docx]

Supplementary Material


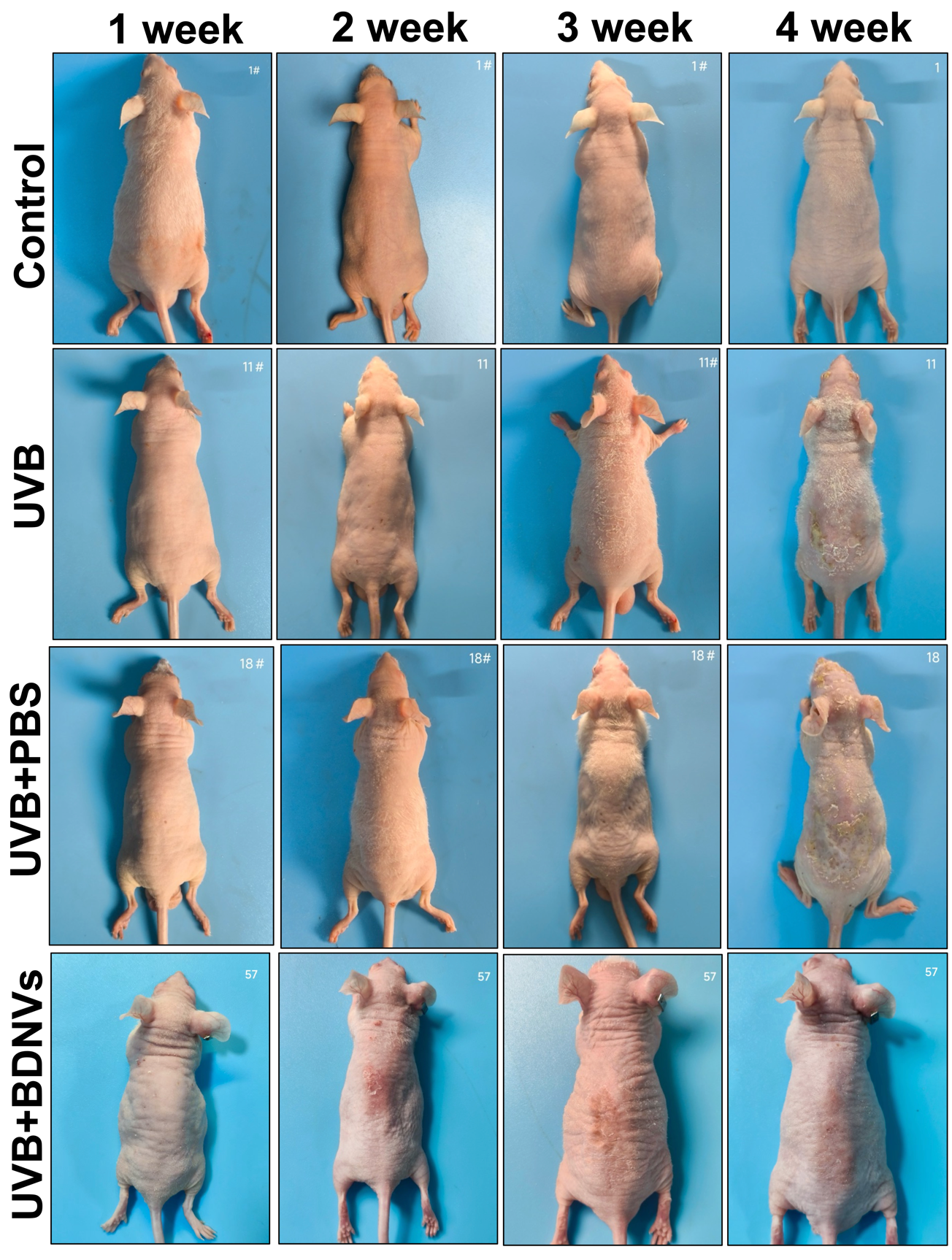


**Figure S1.** Alterations in murine skin tissue following diverse treatments over multiple time points.

**Figure S2**. The influence of BDNVs on the viability of HDFs under different concentration treatments.
